# RNA Oxidation Adducts 8-OHG and 8-OHA Change with Aβ_42_ Levels in Late-Stage Alzheimer's Disease

**DOI:** 10.1371/journal.pone.0024930

**Published:** 2011-09-20

**Authors:** Adam M. Weidner, Melissa A. Bradley, Tina L. Beckett, Dana M. Niedowicz, Amy L. S. Dowling, Sergey V. Matveev, Harry LeVine, Mark A. Lovell, M. Paul Murphy

**Affiliations:** 1 Sanders-Brown Center on Aging, University of Kentucky, Lexington, Kentucky, United States of America; 2 Department of Molecular and Cellular Biochemistry, University of Kentucky, Lexington, Kentucky, United States of America; 3 Department of Chemistry, University of Kentucky, Lexington, Kentucky, United States of America; 4 Department of Molecular and Biomedical Pharmacology, University of Kentucky, Lexington, Kentucky, United States of America; National Institutes of Health, United States of America

## Abstract

While research supports amyloid-β (Aβ) as the etiologic agent of Alzheimer's disease (AD), the mechanism of action remains unclear. Evidence indicates that adducts of RNA caused by oxidation also represent an early phenomenon in AD. It is currently unknown what type of influence these two observations have on each other, if any. We quantified five RNA adducts by gas chromatography/mass spectroscopy across five brain regions from AD cases and age-matched controls. We then used a reductive directed analysis to compare the RNA adducts to common indices of AD neuropathology and various pools of Aβ. Using data from four disease-affected brain regions (Brodmann's Area 9, hippocampus, inferior parietal lobule, and the superior and middle temporal gyri), we found that the RNA adduct 8-hydroxyguanine (8-OHG) decreased, while 8-hydroxyadenine (8-OHA) increased in AD. The cerebellum, which is generally spared in AD, did not show disease related changes, and no RNA adducts correlated with the number of plaques or tangles. Multiple regression analysis revealed that SDS-soluble Aβ_42_ was the best predictor of changes in 8-OHG, while formic acid-soluble Aβ_42_ was the best predictor of changes in 8-OHA. This study indicates that although there is a connection between AD related neuropathology and RNA oxidation, this relationship is not straightforward.

## Introduction

With an increase in the aging population, neurodegenerative diseases are becoming more prevalent. The most common form of dementia in the U.S. is Alzheimer's disease (AD), which is consistently in the top 10 causes of death in the elderly [1]. While the mechanism behind the progression of this disease remains unclear, the importance of amyloid-β (Aβ) as a causative factor in AD is well known [2]. This small peptide is accepted as triggering the initial event that drives the disease [3]. The Aβ peptide is a product of sequential cleavage of the amyloid-β precursor protein (APP), and it has a tendency to aggregate. The 40 amino acid peptide (Aβ_40_) is the most abundant form of Aβ, and is not as aggregate-prone as the less common 42 amino acid peptide (Aβ_42_). The 42∶40 peptide ratio increases in early onset, familial AD [4], indicating that Aβ_42_ may play a greater role in initial pathology than Aβ_40_.

The peptide aggregates become more insoluble as they continue to accumulate - a characteristic that can be discerned by sequential extraction under progressively more denaturing conditions. This allows for categorization of Aβ by solubility, which can help provide insights into the pathophysiology of the disease process [5]. The aggregation process ultimately leads to mature amyloid fibrils which form deposits known as amyloid plaques. Amyloid plaques are found in two different forms: diffuse plaques (DPs) and neuritic plaques (NPs). DPs lack a dense core and are associated with normal aging. NPs possess an amyloid core, associate with dystrophic neurites [6], and increase in AD [7]. NPs, coupled with tangles of hyperphosphorylated tau (neurofibrillary tangles (NFTs)), are the basis of an AD diagnosis in postmortem tissue [8].

Apart from Aβ accumulation, many other factors may contribute to the disease process. An increase in oxidative stress found in the form of oxidized DNA, RNA, and protein adducts may also contribute to the progression of the disease [9], [10]. The occurrence of nucleotide oxidation has the greatest potential for long-term, pathophysiologic consequences, as nucleotide mutations may result in incorrect synthesis of numerous proteins repeated over the life of the cell. For example, 8-hydroxyguanine (8-OHG) can incorrectly base pair with adenine and introduce mutations during DNA synthesis [11]. The effects of oxidative stress on DNA has been focused on more than RNA, even though RNA may be more vulnerable to oxidative insults than DNA given its generally single-stranded state and accessibility to the oxidant-producing mitochondria [12]. RNA oxidation is increased in AD [12], [13] and certain adducts are more abundant than others. The most commonly quantified nucleotide adducts include 8-OHG, 8-hydroxyadenine (8-OHA), 5-hydroxycytosine (5-OHC), 2,6-diamino-4-hydroxy-5-formamidopyrimidine (fapyguanine), and 4,6-diamino-5-formamidopyrimidine (fapyadenine) [14], [15], all of which are found in both DNA and RNA. Determining the role of these adducts in AD is important to understanding the progression of the disease.

Despite studies of RNA oxidation in various neurodegenerative diseases [12], [16], [17], few studies have attempted to relate oxidation with Aβ. Furthermore, variations between studies in methodology, as well as differences in attributes such as post mortem interval (PMI), brain region, and subject population make it difficult to determine the relative importance of different RNA adducts in the progression of the disease, and how these adducts may relate to other aspects of AD neuropathology. In this study, we performed a systematic analysis of multiple forms of Aβ and RNA adducts across several brain regions in a well-characterized cohort of late stage AD cases and non-cognitively impaired control cases.

## Materials and Methods

### Ethics Statement

Human tissue collection and handling conformed to Public Health Service and University of Kentucky Institutional Review Board guidelines, including written informed consent from all participants.

### Tissue Collection

Tissue samples were obtained from the Alzheimer's Disease Center at the University of Kentucky. All subjects were assessed using our standard neuropsychological test battery. Details of subject monitoring have been described previously [18]. Control subjects (N = 10) were age-matched to AD subjects (N = 12), with similar post mortem intervals (PMI) for both groups (see **Table 1** for demographic statistics). Autopsy methods and quantitative neuroanatomy measures have been previously published [19]. Briefly, brain weight was determined at time of autopsy and a neuropathological evaluation was performed. Samples from four disease-affected areas (Brodmann's Area 9, the hippocampal formation, the inferior parietal lobule, and the superior and middle temporal gyri) and one disease-unaffected region (cerebellum) were used for the analysis.

**Table 1:** Summary of Subject Demographic Data.

						Neuropathologic Variables		
	Sex	Age (y)	Brain Weight (g)	Post Mortem Interval (h)	MMSE Score	Neurofibrillary Tangles	Neuritic Plaques	Diffuse Plaques
**Control**	1M/9F	90.0±5.6	1134±118	2.9±0.6	28.0±1.6	1.5±3.8	0.4±1.0	1.3±2.1
**AD**	2M/10F	83.0±6.7	1040±104	3.0±0.7	6.9±7.2*	49.3±17.4*	17.7±5.5*	32.3±11.1*

*All AD cases were Braak stage VI; control cases were all stage II or less. Neuropathologic variables were averaged across several disease affected brain regions (inferior parietal lobule, midfrontal gyrus, superior and middle termporal gyri, hippocampal area CA1, and the subiculum). Values are mean +/− standard deviation;*

* = p<0.01 relative to control cases, adjusted for multiple comparisons.

### Measurement of Aβ

We used a well-known method of serial extraction and quantification of Aβ based on solubility [20]. Briefly, tissue was homogenized via polytron in PBS (pH 7.4; 1.0 mL/200 mg wet tissue weight) with a complete protease inhibitor cocktail (PIC, Amresco; Solon, OH) and centrifuged at 20,800× *g* for 30 min at 4°C. The supernatant (PBS-soluble pool) was collected and the remaining pellet was sonicated (10×0.5 sec pulses at 100 W, Fisher Sonic Dismembrator, Fisher; Pittsburgh, PA) in 2% (w/v) SDS with PIC and centrifuged as above at 14°C. This supernatant (SDS-soluble pool) was collected, and the pellet was sonicated a final time in 70% (v/v) formic acid, and then centrifuged at 20,800× *g* for 60 min at 4°C. The remaining supernatant fraction was collected (FA-soluble pool). All samples were stored at −80°C until time of assay.

Quantification of total Aβ was performed using a two-site sandwich ELISA [21], whereas oligomeric Aβ was quantified using a single-site sandwich ELISA (4G8/4G8) [22]. Immulon 4HBX plates (Nunc; Rochester, NY) were coated with 0.5 µg/well antibody Ab9 (Aβ 1–16, two-site ELISA) or 4G8 (Aβ 17–24, single-site ELISA; Covance; Princeton, NJ) and incubated overnight at 4°C [23], [24]. Wells were blocked with Synblock® (AbD Serotec; Oxford, UK) according to manufacturer's directions. Standard curves were prepared using synthetic Aβ peptide (rPeptide; Bogart, GA). PBS samples were diluted 1∶4 in Antigen Capture (AC) buffer (20 mM Na_3_PO_4_, 0.4% Block Ace (AbD Serotec), 0.05% NaN_3_, 2 mM EDTA, 0.4 M NaCl, 0.2% BSA, 0.05% CHAPS, pH 7). SDS samples were diluted between 1∶20 and 1∶100 in AC buffer, and formic acid samples were first neutralized 1∶20 in TP buffer (1.0 M Tris base, 0.5 M Na_2_HPO_4_) then further diluted between 1∶5 and 1∶20 in AC buffer. Standards and samples were run at least in duplicate and were incubated at 4°C overnight. Biotinylated detection antibodies were Ab13.1.1 (Aβ_40_-end specific), 12F4 (Aβ_42_-end specific; Covance) or 4G8 (Aβ_17–24_), followed by the addition of NeutrAvidin-HRP (Pierce Biotechnologies; Rockford, IL). Colorimetric detection used 3,3′,5,5′-tetramethylbenzidine reagent (TMB; Kirkegaard & Perry Laboratories; Gaithersburg, MD). The reaction was stopped via acidification (6% *o*-phosphoric acid) and read using a BioTek Powerwave XS (Winooski, VT) plate reader at 450 nm.

Pittsburgh compound B (PiB) binding in post-mortem human specimens has been used to detect fibrillar Aβ, the most advanced form of Aβ aggregation [25]. The degree of PiB binding may differentiate between detrimental and benign fibrillar Aβ aggregates [26]. Quantification of PiB binding in the PBS homogenate was performed as described by Rosen et al. [26]. Homogenate (5 µl) was added to 250 µl PBS in a 96-well polypropylene plate on ice. Twenty microliters of this diluted homogenate was then transferred to another plate in triplicate. In two wells, 200 µl of 1.2 nM 3H-PIB in PBS+5% (v/v) EtOH was added to measure total binding. In the third well, 1.2 nM 3H-PIB in PBS+5% (v/v) EtOH plus 1 µM BTA-1 was added to correct for non-specific binding. The contents of the plate were transferred to a Multiscreen-FB (GF/B) (Millipore; Billerica, MA) plate and washed with PBS. The glass fiber filters were soaked overnight in Budget-Solve (RPI; Mount Prospect, IL) scintillation fluid, counted, and standardized to the BTA-containing wells.

### RNA isolation and Sample Preparation

RNA isolation was performed using TRIzol® (Invitrogen; Carlsbad, CA) as described in Rio et. al. [27]. Briefly, tissue was homogenized in TRIzol® (1 mL/100 mg; Invitrogen) as per manufacturer's instructions, followed by an additional phenol-chloroform extraction and ethanol precipitation.

To prepare samples for gas chromatography (GC), we used a procedure similar to that described in Wang et al. [28]. RNA (10–20 µg) was analyzed by GC/mass spectrometry (GC/MS) with selective ion monitoring (see below). Stable-labeled oxidized base internal standards (10 nmol) including 8-[8-^13^C,7,9-^15^N_2_] hydroxyguanine, 8-[8-^13^C,6,9-diamino-^15^N_2_] hydroxyadenine, 5-[2-^13^C,1,3-^15^N_2_] hydroxycytosine, [formyl-^13^C, diamino-^15^N_2_] fapyadenine and [formyl-^13^C, diamino-^15^N_2_] fapyguanine (Cambridge Isotope Laboratories; Andover, MA) were added to lyophilized RNA samples and were hydrolyzed with 250 µL 90% formic acid at 145°C for 30 min in evacuated 5 mL conical glass tubes. After hydrolysis, samples were lyophilized and derivatized with a mixture of *N*,*O*-bis-trimethylsilyltrifluoroacetamide/pyridine (1∶1) at 25°C for 2 h in evacuated tubes. The derivatized products were dried under a constant stream of nitrogen using an OA-SYS heating system (Organomation Associates; Berlin, MA). Derivatized RNA base adducts were dissolved in 20 µL *N*,*O*-bis-trimethylsilyltrifluoroacetamide and transferred to GC autosampler vials.

### Gas chromatography/mass spectrometry with selective ion monitoring analysis

Derivatized samples (2 µL) were analyzed using an Agilent 7800A gas chromatograph (Agilient Technologies; Santa Clara, CA) on an HP 5 ms capillary column (0.25 mm internal diameter, 0.25 µm film thickness, and 30 m length; Hewlett Packard, Palo Alto, CA, USA) as previously described [28]. Chromatographic parameters were as follows: ultra high purity helium was used as a carrier gas at an inlet pressure of 11.8 psi, and used constant flow and split-less mode. The injection port was maintained at 250°C. The initial temperature was held for 2 min at 100°C after sample injection with the following ramps: ramp 1, 100–178°C at 3°C/min; ramp 2, 178–181°C at 0.3°C/min; ramp 3, 181–208°C at 3°C/min and ramp 4, 208–280 at 10°C/min. The final temperature was maintained for 2 min, the run time was 56.2 min for each sample, and the temperature of the ion source inside the mass spectrometer was 180°C. Derivatized nitrogenous base spectra were acquired in selective ion monitoring mode at m/z ratios of 331 5-[2-^13^C,1,3-^15^N_2_] hydroxycytosine and m/z 328 5-hydroxycytosine; m/z 357 [formyl-^13^C, diamino-^15^N_2_] fapyadenine and m/z 354 fapyadenine; m/z 355 8-[8-^13^C,6,9-diamino-^15^N_2_] hydroxyadenine and m/z 352 8-hydroxyadenine; m/z 445 [formyl-^13^C, diamino-^15^N_2_] fapyguanine and 442 fapyguanine: and m/z 443 8-[8-^13^C,7,9-^15^N_2_] hydroxyguanine and m/z 440 8-hydroxyguanine. Instrument response plots of the integrated peaks of stable isotope-labeled analyte signal added were determined over a range of 0.5 nmol to 10.0 nmol per stable isotope-labeled analyte. Plots of instrument response versus concentration showed positive significant correlations for stable label isotopes: 5-[2-^13^C,1,3-^15^N_2_] hydroxycytosine (*r = *0.94): [formyl-^13^C, diamino-^15^N_2_] fapyadenine (*r* = 0.97): 8-[8-^13^C,6,9-diamino-^15^N_2_] hydroxyadenine (*r* = 0.91); [formyl-^13^C, diamino-^15^N_2_] fapyguanine (*r* = 0.92); 8-[8-^13^C,7,9-^15^N_2_] hydroxyguanine (*r* = 1.00). The integrated area of each analyte signal was normalized with respect to the integrated area of the corresponding internal standards for all samples and corrected based on instrument response plots.

### Data Analysis

**Figure 1:**
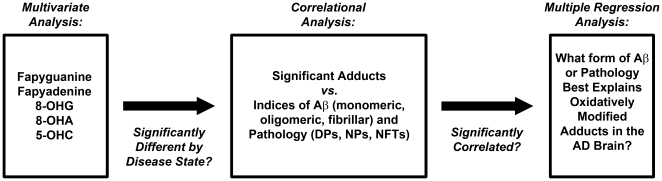
Flowchart depicting the steps comprising the data analysis. A directed analysis of the data was used to reduce the large data set to its significant components. A multivariate analysis determined which variables were significantly different in AD using PMI and age as covariates and gender as a variable; only significant variables were included in further analysis. A Spearman correlation was used to determine the relationships between the five RNA adducts of interest and the other measures of AD pathology. Finally, these significant variables were analyzed by stepwise multiple regression to determine if they predicted the changes in the RNA adducts.

We used a reductive approach to determine relative significance (**Figure 1**). A Spearman correlation was used to determine the relationships between the five RNA adducts and the other measures of pathology across multiple brain regions. These pathology measures included NFTs, DPs, NPs, PiB binding (fibrillar Aβ), Aβ oligomers, and the various soluble pools of Aβ. A multivariate analysis (which included gender as a variable, and both PMI and age as covariates) was performed to determine which variables were significantly different in the AD-affected individuals. These variables were analyzed by stepwise multiple regression to determine if any variable or combination of variables were associated with changes in the RNA adducts of interest. Known differences in group demographics were verified by Student's *t*-test or Mann-Whitney U-test where appropriate, adjusting for multiple testing using the Holm-Bonferroni method [29]. All data were analyzed using PASW® 18 (IBM; Somers, NY). Graphs and regression lines were calculated and produced in SigmaPlot® (Systat; San Jose, CA).

## Results and Discussion

While oxidatively modified RNA is increased in AD [12], [13], when compared to both oxidatively modified DNA and protein, RNA is understudied. However, RNA may be a better marker of oxidative stress (*e.g.* more susceptible to damage) in the AD brain. In spite of several studies documenting changes in RNA oxidation in the AD brain, it is not clear which adducts are more important. It can be difficult to compare reported results because of variations in methods, tissue sample quality, and brain regions among separate studies. In this study, we took a systematic approach to answer these questions.

Using GC/MS, we quantified five forms of oxidatively modified RNA in the AD brain (8-OHG, 8-OHA, 5-OHC, fapyadenine, and fapyguanine). We analyzed the relationship between these RNA adducts and the common neuropathological markers used to diagnose AD (neuritic plaques, diffuse plaques, and neurofibrillary tangles). A correlational analysis of the data did not indicate any significant relationships between any of these markers and any of the five RNA adducts (p>0.1 in all cases). This was true across all cases, even when the analysis was restricted to AD cases alone. Therefore, although all three markers of neuropathology are significantly higher in the AD cases, they are independent of RNA oxidation.

**Figure 2:**
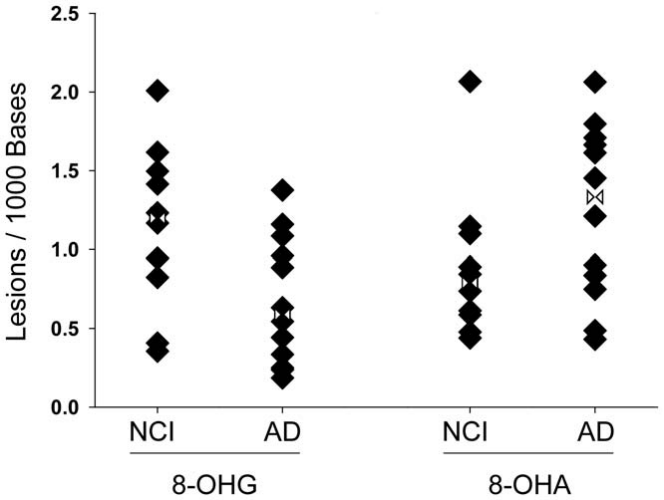
8-OHG and 8-OHA change in opposite directions in the late-stage AD brain. The 8-OHG adduct decreased (p = 0.046) in the disease state, whereas the 8-OHA adduct increased in AD brain (p = 0.038). Data are expressed as the number of oxidatively modified bases per 1000 bases of total RNA. The analysis included gender, age and PMI. Values were averaged over several disease-affected brain regions (*c.f.* Table 1); no significant changes were seen in cerebellum. NCI: No Cognitive Impairment; AD: Alzheimer's disease. Rotated Hourglass: mean.

We performed a multivariate analysis to determine if any of the RNA adducts differed between AD cases and controls. We did not detect any difference in RNA adducts between disease states in the cerebellum. This was not unexpected, as the cerebellum does not typically exhibit substantial pathology in AD. An analysis restricted to the disease- affected regions (Brodman's Area 9, hippocampus, inferior parietal lobule, and the superior and middle temporal gyri) showed that two of the five RNA adducts changed in AD cases when compared to controls (**Figure 2**). 8-OHA lesions increased [F(1,16) = 5.12, p<0.04], whereas 8-OHG decreased [F(1,16) = 4.66, p<0.05].

Abundant oxidation of guanine is attributed to its high oxidation potential. However, the oxidation potential of guanine has been shown to be modulated based on location (5′ vs. 3′), stacking base interactions, existing mismatched base pairing, and flanking nucleic acid sequence in double-stranded DNA [30], [31], [32]. To date, modulation of oxidation potentials have not been evaluated in an RNA model similar to polyadenylated mRNA. The possibility of modulated oxidation potentials and the abundance of adenine may contribute to the dichotomy observed in these RNA adducts. Alternatively, this may represent a temporal process, whereby 8-OHG accumulates earlier during the disease and then declines, while 8-OHA increases later in the disease. Evidence from Nunomura et. al. [31] suggests Aβ accumulation directly reduces oxidative adducts, resulting in decreased 8-OHG levels with increased pathology. Since the current study only includes late stage AD cases, answering this question would require an examination of multiple RNA adducts across several brain regions in very early or preclinical AD cases.

As expected, a multivariate analysis showed increased total Aβ in AD, in all regions except the cerebellum [F(1,16) = 5.08, p<0.04]. The amount of post-mortem PiB binding was also significantly increased [F(1,16) = 17.84, p<0.001]. Surprisingly, we did not find higher total amounts of oligomeric Aβ in the AD cases compared to the control cases [F(1,16) = 2.45, p<0.14]. This was unexpected, since there is typically an abundance of oligomeric Aβ in the AD brain, which can be visualized by a variety of methods [25], [33]. Since our assay primarily measures larger oligomers (>40 kDa) [22], this may indicate that the increase in oligomeric Aβ in AD is mostly smaller forms that we do not detect. Aβ oligomers cause cell death and changes in morphology and function [34], [35], and recent reports show that Aβ dimers may play a major role in AD pathophysiology [36]. Further study is required to assess the relative contribution of smaller oligomeric species to RNA oxidation in the AD brain.

Aβ has been suggested as a prime etiological agent of AD, and thus may relate to or even drive nucleotide oxidation [2]. We performed a stepwise multiple regression analysis to determine if any pools of Aβ were linked to changes in the two RNA adducts (8-OHA and 8-OHG). For the analysis, we deconvoluted the total Aβ measure to its individual component measures (PBS-, SDS- or FA-soluble Aβ_40_ and Aβ_42_) to identify the major contributor to disease-associated variance. We have shown previously that the solubility of the Aβ peptide is an important variable in AD [20], [37]. The PBS-soluble fraction primarily contains monomeric and low-weight Aβ oligomers, the SDS-soluble fraction contains higher order Aβ multimers and is associated with the amount of DPs, and the FA-soluble fraction contains highly insoluble accumulated Aβ associated with NPs [20].

**Figure 3:**
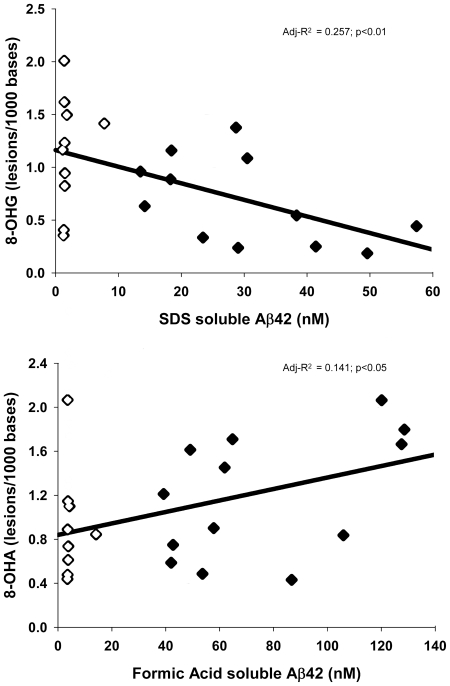
Oxidation adducts are modeled by single but separate pools of Aβ. Using multiple stepwise regression, the decrease in 8-OHG was correlated to SDS-soluble Aβ_42_ (*top*; adj-R^2^ = 0.257; p<0.01) while the increase in 8-OHA lesions was correlated to formic acid-soluble Aβ_42_ (*bottom*; adj-R^2^ = 0.141; p<0.05). Data are expressed as the number of oxidatively modified bases per 1000 bases of total RNA. Open Symbol: NCI; Closed Symbol: AD.

Neither fibrillar Aβ, as measured by PiB binding, nor any pools (PBS-, SDS- or FA-soluble) of Aβ_40_ were significantly related to the changes in 8-OHA or 8-OHG in AD. This was not unexpected, as the Aβ_40_ species is generally accepted as the less toxic form, and is less implicated in the disease process. However, the disease-related changes in the two RNA adducts were associated with SDS-soluble and FA-soluble levels of Aβ_42_: SDS-soluble Aβ_42_ was inversely related to 8-OHG [F(1,20) = 8.28,p<0.01] and FA-soluble Aβ_42_ was directly related to 8-OHA [F(1,20) = 4.44,p<0.05] (**Figure 3**). As might be expected from the Spearman correlational analysis, no form of Aβ predicted the amount of the other three RNA adducts (fapyadenine, fapyguanine, or 5-OHC) in the brain; AD neuropathologic markers (DPs, NPs, NFTs) also did not predict the amount of these adducts.

The reason behind the opposite directionality of these adducts is unclear. However, the fact that they relate to different soluble levels of Aβ_42_ may indicate that the adducts form at different stages of AD progression, as the amount of FA-soluble Aβ increases more in late stage disease [20]. Of the disease-affected brain regions studied, the RNA adducts in the hippocampus had the strongest correlation to disease effect. The hippocampus is an early structure which is affected in the disease process, and exhibits severe atrophy in later stages of the disease [38]. It is possible that 8-OHG increases here initially in the disease, but then declines. If this is true, then an evaluation of an expanded cohort of subjects in earlier disease states such as preclinical AD and mild cognitive impairment would be needed to confirm this.

Of the five RNA adducts analyzed, only 8-OHG and 8-OHA were significantly altered in late stage AD, and each related to a single extractable pool of Aβ_42_: 8-OHG was related to SDS-soluble Aβ_42_, and 8-OHA was related to FA-soluble Aβ_42._ These adducts were not related to other pools of Aβ nor to any standard neuropathological markers of AD. Interestingly, while 8-OHA increased in late-stage AD, 8-OHG decreased. It is possible that the different behavior of the two RNA adducts is explained by a temporal mechanism. Our examination of only non-cognitively impaired subjects and late-stage AD cases omits a time span where an increase in 8-OHG may occur and then decline. Indeed, 8-OHG is increased in several disease affected regions in earlier stage AD [39], including the hippocampus [40]. The decrease of 8-OHG may be explained by the extensive cell death which occurs in late-stage AD, although this can be difficult to deal with analytically [41], [42]. The decrease may also be a result of the direct influence of Aβ as a compensatory mechanism [31]. This work raises several interesting avenues for mechanistic studies. The specific relationship between toxic Aβ aggregates and problematic RNA derivatives remains to be explored.
